# Joint analyses of multi-tissue Hi-C and eQTL data demonstrate close spatial proximity between eQTLs and their target genes

**DOI:** 10.1186/s12863-019-0744-x

**Published:** 2019-04-30

**Authors:** Jingting Yu, Ming Hu, Chun Li

**Affiliations:** 1 Department of Population and Quantitative Health Sciences, Case Western Reserve University, Cleveland, OH USA; 2 Department of Quantitative Health Sciences, Lerner Research Institute, Cleveland Clinic Foundation, Cleveland, OH USA; 3 Cleveland Institute for Computational Biology, Cleveland, OH USA

**Keywords:** Hi-C, eQTL, Gene regulation, Primary human tissues, Human cell lines

## Abstract

**Background:**

Gene regulation is important for cells and tissues to function. It has been studied from two aspects at the genomic level, the identification of expression quantitative trait loci (eQTLs) and identification of long-range chromatin interactions. It is important to understand their relationship, such as whether eQTLs regulate their target genes through physical chromatin interaction. Although chromatin interactions have been widely believed to be one of the main mechanisms underlying eQTLs, most evidence came from studies of cell lines and yet no direct evidence exists for tissues.

**Results:**

We performed various joint analyses of eQTL and high-throughput chromatin conformation capture (Hi-C) data from 11 human primary tissue types and 2 human cell lines. We found that chromatin interaction frequency is positively associated with the number of genes that have eQTLs and that eQTLs and their target genes tend to fall into the same topologically associating domain (TAD). These results are consistent across all tissues and cell lines we evaluated. Moreover, in 6 out of 11 tissues (aorta, dorsolateral prefrontal cortex, hippocampus, pancreas, small bowel, and spleen), tissue-specific eQTLs are significantly enriched in tissue-specific frequently interacting regions (FIREs).

**Conclusions:**

Our data have demonstrated the close spatial proximity between eQTLs and their target genes among multiple human primary tissues.

**Electronic supplementary material:**

The online version of this article (10.1186/s12863-019-0744-x) contains supplementary material, which is available to authorized users.

## Background

Gene regulation is important for cells and tissues to function. Differences in gene regulation are often responsible for cellular and morphological differences between cell lines and tissues. The advancement of high-throughput technologies such as DNA and RNA sequencing and SNP chips allows researchers to study gene regulation at the genomic level and from multiple perspectives. On the one hand, motivated by the likely functional importance of genetic variants in gene regulation, many studies have focused on identifying expression quantitative trait loci (eQTLs), which are genetic variants statistically associated with gene expression across individuals [1–4]. eQTLs can regulate the expression of their target genes by altering *cis*-regulatory elements (CREs) such as enhancers, promoters, insulators, mediators, etc. [5–7]. On the other hand, analyses of chromatin spatial organization have established the importance of chromatin interaction in gene regulation [8–10]. For example, by forming long-range chromatin interactions, CREs can regulate the expression of their target genes hundreds of kilobases (Kb) away [11–13]. High-throughput chromatin conformation capture (Hi-C) has been widely adopted to provide a genome-wide view of chromatin interactions within a tissue or cell line [14–17]. Hi-C data are usually presented as a chromatin contact matrix, in which the genome is divided into equal-sized bins. The value of each element in the matrix represents the number of read pairs mapped to a pair of bins, which is called the chromatin interaction frequency (CIF).

These two complementary approaches focus on different aspects of gene regulation. eQTL results are statistical across individuals and require an associated SNP, while chromatin interactions are physical within a sample and do not require a polymorphism to be present. It is desirable to integrate the results of these two approaches to better understand their relationships, such as whether eQTLs regulate their target genes through chromatin interactions. Analyzing Hi-C data from human IMR90 fibroblasts and embryonic stem cells, Duggal et al. [18] showed that eQTLs are spatially close to their target genes, especially for those located within the same topologically associating domain (TAD) and overlapping with CREs, and that genomic regions containing eQTLs tend to have a higher CIF. Consistent with Duggal et al., using Hi-C data from human cell lines generated by Rao et al. [14], the Genotype-Tissue Expression (GTEx) study [3] has also shown that eQTLs that are enriched for CREs are in close spatial proximity with their target gene promoters. In both studies, the Hi-C data came only from cell lines, while the eQTL results were generated from both tissues and cell lines. Some other studies, for example, those aimed to predict enhancer-promoter interactions [19–21] and those aimed to detect regulatory SNPs [22], did not provide evidence to support the relationship between Hi-C data and eQTL results, but instead used this connection as ground truth to demonstrate the validity of their own results.

Although chromatin interactions have been widely believed as one of the main mechanisms underlying eQTLs, we are unaware of any direct evidence of this for tissues. It is well known that eQTLs are tissue specific [3]. Moreover, Schmitt et al. [23] recently identified hotspots of local chromatin interactions from Hi-C data, called frequently interacting regions (FIREs). FIREs are bins that frequently interact with nearby regions <200Kb, and they display strong tissue specificity. It is unclear how much overlap exists between tissue-specific FIREs and tissue-specific eQTLs.

**Fig. 1:**
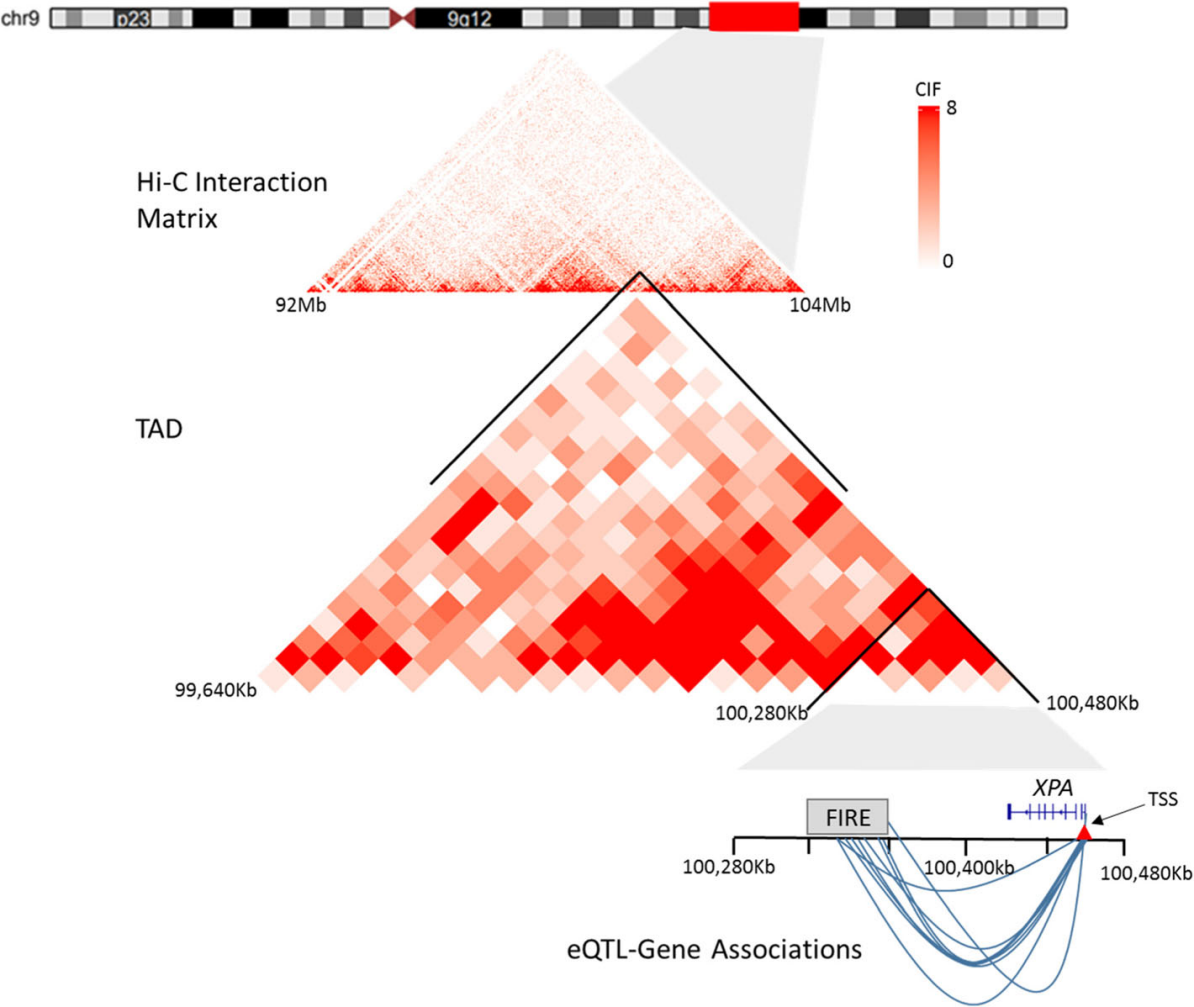
An example to show the connections between Hi-C data and eQTL results. The top triangle is the raw Hi-C contact matrix for a 12 Mb region in chromosome 9 (chr9:92,000,000-104,000,000) for the DLPFC tissue at 40Kb resolution. The bottom triangle shows a TAD (chr9:99,640,000-100,480,000), which contains a DLPFC-specific FIRE (chr9:100,320,000-100,360,000) and an eGene, *XPA* (xeroderma pigmentosum complementation group A; chr9:100,437,191-100,459,639). The GTEx study identified 20 eQTLs inside this FIRE for *XPA* in the tissue of brain frontal cortex

Hi-C and eQTL data are now available for multiple human primary tissues and cell lines. For example, Schmitt et al. [23] generated Hi-C data for 14 human primary tissues and 7 human cell lines, and the GTEx study [3] performed genome-wide mapping of eQTLs across 48 human tissues. There are 11 tissues and 2 cell lines that overlap between these two sources (See Additional file 1: Supplementary Materials and Table S1), facilitating a direct evaluation of the relationship between chromatin interactions and eQTLs across multiple tissues and cell lines. Fig. 1 shows an example of the connection between Hi-C data and eQTL results.

We performed a series of joint analyses on the relationship between Hi-C data and eQTL results. We found that CIF is positively associated with the number of eGenes identified from the GTEx study (an eGene is defined as a gene in which the expression is significantly associated with an eQTL), and that eQTLs and their target genes are more likely to co-localize within the same TAD than randomly generated control datasets. All these results are consistent across all tissues and cell lines we evaluated. Since both eQTLs and FIREs are known to be highly tissue specific [3, 23], we also studied the relationship between tissue-specific eQTLs and tissue-specific FIREs and found that majority of the tissues demonstrate a positive association between them.

To the best of our knowledge, our study is the first to demonstrate the relationship between chromatin interactions and eQTLs across multiple human primary tissues, and to study the relationship between tissue-specific eQTLs and tissue-specific FIREs. These results help improve our understanding of the roles of chromatin interactions and eQTLs in gene regulation mechanisms.

## Results

### Chromatin interaction frequency is positively associated with the number of eGenes

**Fig. 2:**
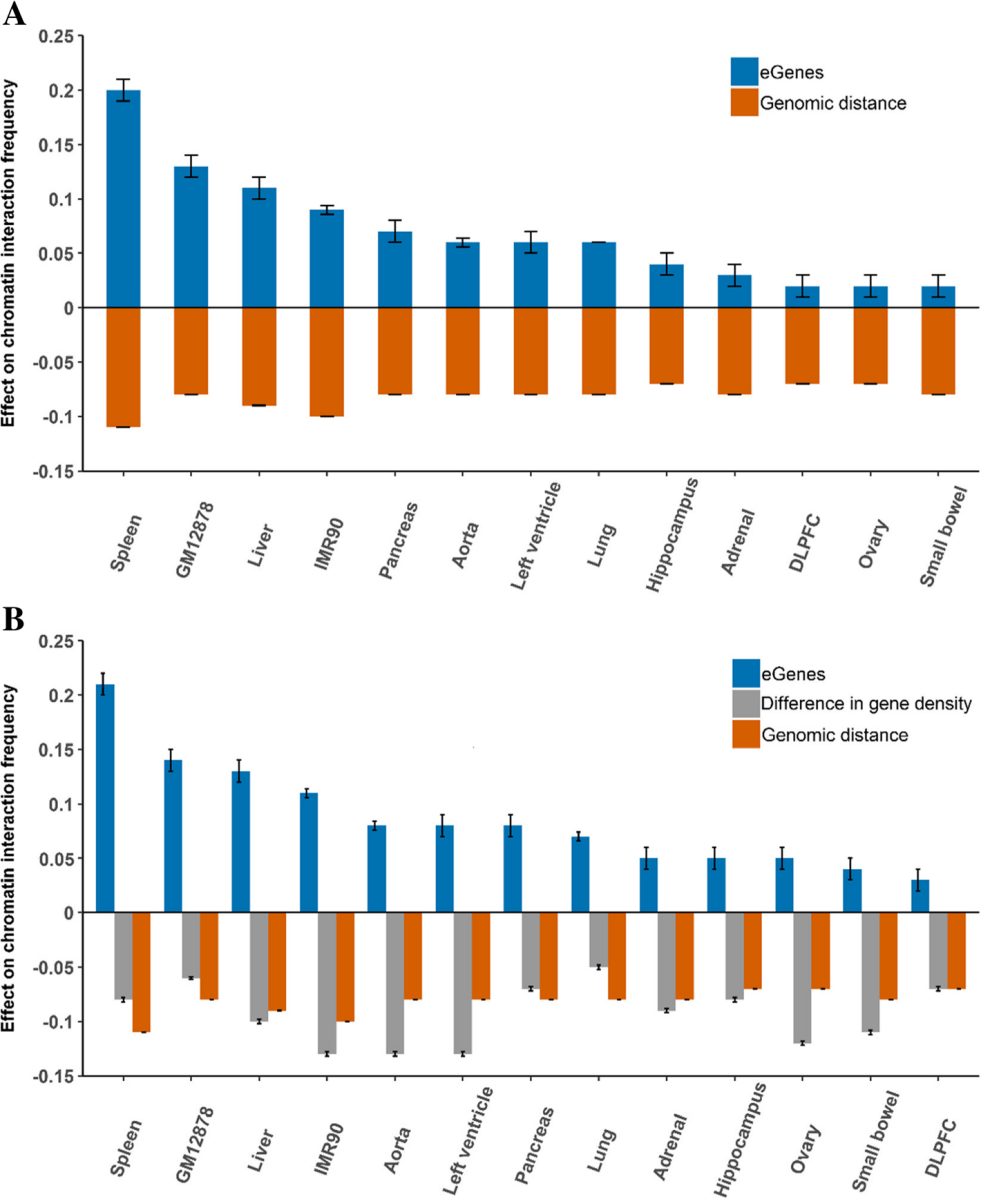
Effect of the number of eGenes on CIF. Two negative binomial regression models were fitted to estimate the effect of the number of eGenes (blue) on CIF, one with adjustment for genomic distance (orange) (**a**), and the other with adjustment for both genomic distance (orange) and the difference in gene density (grey) (**b**). The error bars are ± standard error

If chromatin spatial organization affects how eQTLs regulate their target genes, one would expect that a pair of genomic loci mapped with eQTL-gene associations would interact frequently. To test this hypothesis, we fitted negative binomial regression models to evaluate the relationship between the number of eGenes and CIF between two loci at the 40Kb bin resolution. In our analysis, we only considered chromatin interactions between different bins, and eQTL-gene pairs that fall into different bins (see Methods). After adjusting for genomic distance between loci, the number of eGenes showed significantly positive effects on CIF across all tissues and cell lines (Fig. 2a). For example, in spleen, the effect of the number of eGenes is estimated to be 0.20 (*p* value <2.2 x 10 − 16), indicating that CIF would be 1.22 ( = *e*^0.20^) times higher for every extra eGene in a 40Kb bin pair. The magnitude of the effects varies across tissues and cell lines, ranging from 0.02 to 0.20. As expected, the effect sizes are similar for tissues from the same organ, such as the two brain tissues, DLPFC and hippocampus. Moreover, genomic distance has a significant negative effect on chromatin interaction and the effects are similar across all tissues and cell lines. This is expected because CIF between two genomic regions tends to decrease as their genomic distance increases [16].

Lieberman-Aiden et al. [16] have discovered the A and B compartments, which are associated with relatively high and low gene density, respectively, and showed that two regions within the same type of compartment (A vs. A or B vs. B) have a higher CIF than regions within different types of compartment (A vs. B). This result indicates that gene density may play a role in Hi-C interaction, and motivated us to add the absolute difference in the number of tested genes between the two bins as a covariate in our regression model (Fig. 2b). As expected, the difference in gene density has a significant negative effect on CIF in all tissues and cell lines. The difference in gene density also has little correlation with the number of eGenes (Pearson correlation ≤0.06 in all tissues and cell lines). Moreover, the number of eGenes is still positively correlated with CIF, and its estimated effects are slightly increased compared to the model without the difference in gene density. Furthermore, we also stratified the data by the difference in gene density (Additional file 1: Figure S1). The difference in the number of eGenes varied from 0 to 8 and the sample sizes were relatively small for the strata with difference > 5 (Additional file 1: Table S2). For most strata, the results showed similar patterns as those in Fig. 1; in some strata, the estimated effects for the number of eGenes were negative but they were not statistically significant (Additional file 1: Figure S1).

We also repeated the analyses with the number of eGenes replaced by the number of genes without any associated eQTL and by the number of genes not expressed in the corresponding tissue or cell line (Additional file 1: Figure S2). The latter two had mostly opposite effects from the number of eGenes. For spleen and GM12878, the effects of the number of genes without eQTL were positive, but at a much smaller magnitude than those for the number of eGenes. These results clearly demonstrate that CIF is associated with the number of eGenes, not the total number of genes.

To ensure the conclusions are not sensitive to the choice of models, we also performed alternative analyses by taking the log-transformation of covariates (see Methods). The results still support the conclusion of positive association between CIF and the number of eGenes (Additional file 1: Supplementary Materials).

### eQTL-gene associations are enriched in TADs

**Fig. 3:**
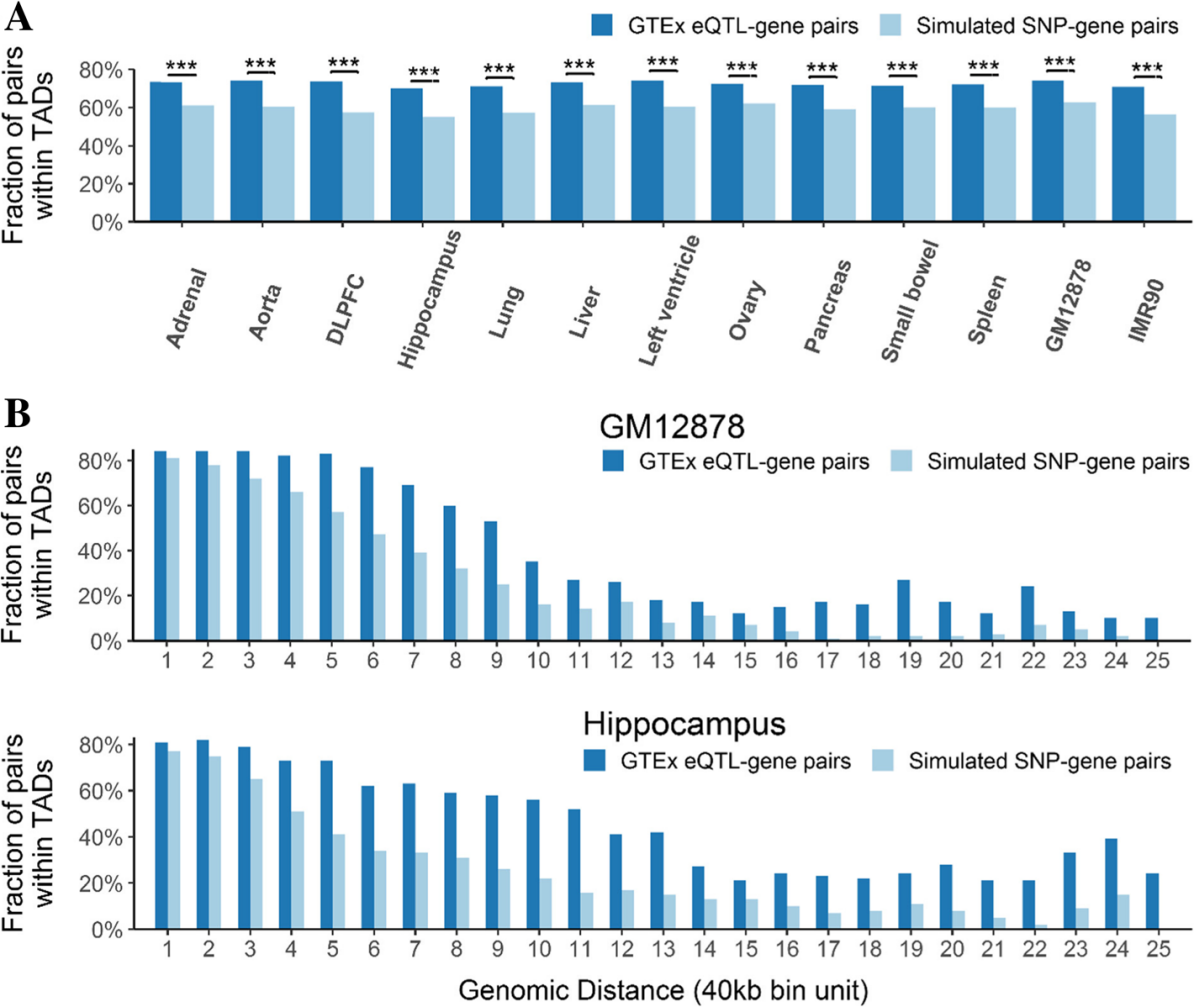
Enrichment of eQTL-gene associations in TADs. (**a**) The fraction of SNP-gene pairs within TADs for the real data (dark blue) and the simulated data (light blue). (***) *p* < 0.001 for Fisher’s exact test. (**b**) Two examples, GM12878 and hippocampus, showing more detailed results after stratifying by the distance between eQTL and TSS of target genes (x-axis, in 40Kb). All comparisons have *p* < 0.001

Since genomic regions within the same TAD are known to interact more frequently than those in different TADs [24, 25], we next examined whether eQTL-gene associations are enriched within TADs. For each tissue and cell line, we simulated a pseudo SNP-gene pair to match every real eQTL-gene pair by keeping the location of the TSS of the gene but flipping the SNP position to the opposite side of the TSS (details in Methods). Most eQTL-gene pairs stayed in the same TAD after flipping, but a significant number of them changed from being inside the same TAD to falling in different TADs (range 10–15% across tissues and cell lines), while none changed the other way (McNemar’s test *p*-value < 2.2 × 10^− 16^ for all tissues and cell lines). The real data also had a significantly higher fraction of eQTL-gene pairs falling in the same TAD than the simulated data (Fisher’s exact test p-value < 2.2 × 10^− 16^ for all tissues and cell lines; Fig. 3a). For example, 74.0% of the real eQTL-gene associations and 62.8% of the simulated pairs in GM12878 were inside TADs.

**Fig. 4:**
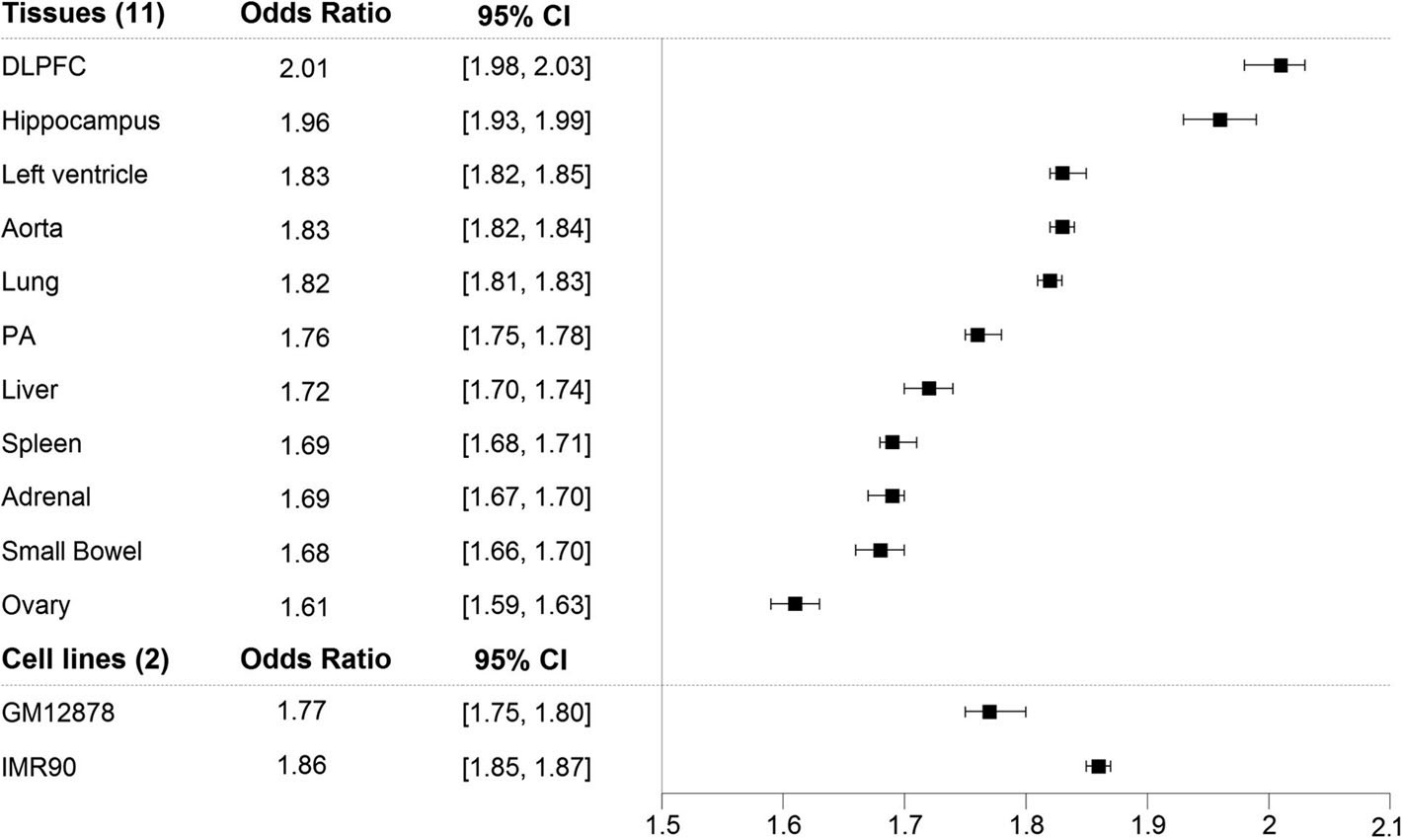
Odds ratio and 95% confidence interval (CI) for SNP-gene pairs mapping at the same TAD between real and pseudo data

The associations also hold when we take the genomic distance into account, both in a joint analysis with genomic distance as a covariate and in stratified analyses with genomic distance as the stratifying variable (see Methods). In the logistic regression, the odds for a SNP-gene pair to be in the same TAD is significantly higher for the real data than for the pseudo data, and the results are consistent across all tissues and cell lines (Fig. 4). The results for our stratified analyses are in Fig. 3b (for GM12878 and hippocampus) and Additional file 1: Figure S4.

### Tissue-specific eQTLs are enriched in tissue-specific FIREs in the majority of tissues

**Fig. 5:**
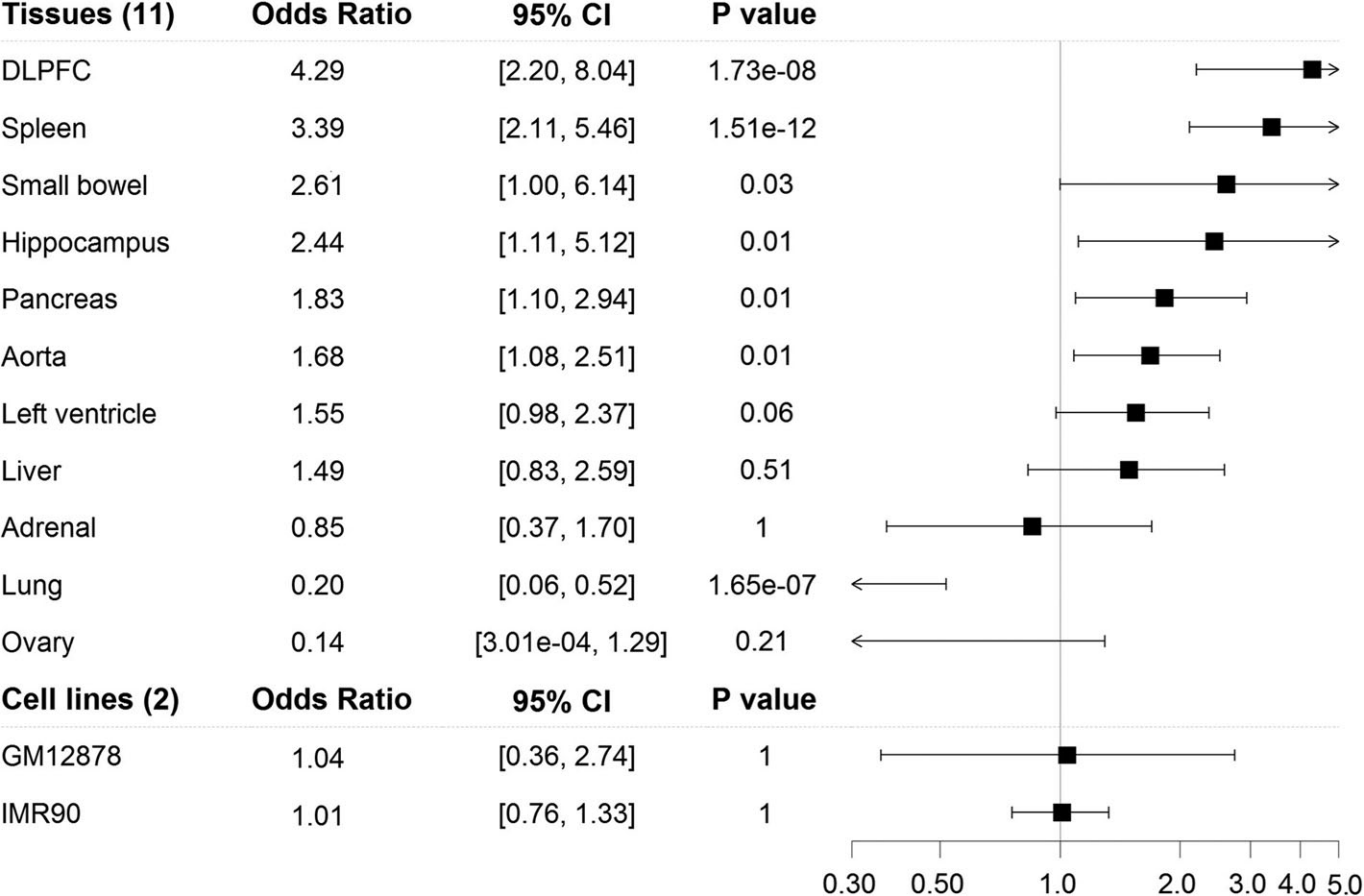
Enrichment of tissue-specific eQTLs in tissue-specific FIREs. We estimated odds ratio, 95% confidence interval after Bonferroni correction, and *p*-value after Bonferroni correction from Fisher’s exact test. The lower bound for small bowel is 1.002

Since eQTLs also have high tissue specificity [3], we then examined whether tissue-specific eQTLs are enriched in tissue-specific FIREs. For the 11 tissues we considered, a total of 349,311 eQTLs were tissue specific. Eight (73%) of the 11 tissues had estimated odds ratio > 1, indicating enrichment of tissue-specific eQTLs in tissue-specific FIREs. Among them, six (55%) tissues (DLPFC, spleen, small bowel, hippocampus, pancreas and aorta) were statistically significant after Bonferroni correction (Fig. 5), and left ventricle almost met the cutoff. This significant enrichment of tissue-specific eQTLs in tissue-specific FIREs suggest that tissue-specific eQTLs may function through chromatin interactions that are also tissue specific, at least in the six tissues that were significant. However, a significant negative association was found in lung. There could be multiple reasons for this result: 1) a more complicated relationship might exist between eQTLs and chromatin spatial organization in lung than in some other issues; 2) the biospecimen used to generate the eQTL and Hi-C data were collected from different people and might have been sampled from different locations of the lung; 3) those samples might be heterogeneous, consisting of different ratios of cell types. Further experimental data will be needed to help evaluate these potential factors. The results for other tissues and the two cell lines were not significant (Fig. 5). Although the results did not have a consistent direction across all tissues, they did suggest that more tissues may have a positive association between tissue-specific eQTLs and tissue-specific FIREs than a negative association.

## Discussion

Chromatin spatial organization and eQTLs are known to be involved in gene regulation. In this work, we systematically studied the relationship between eQTL-gene associations and chromatin interactions across 11 tissues and 2 cell lines. We found that CIF is positively associated with the number of eGenes. Moreover, we found that eQTL-gene associations are enriched in TADs. These results hold in all tissues and cell lines we evaluated. While these results may have been widely expected to hold, we have now provided solid statistical evidence across multiple tissues. These results suggest that eQTLs may regulate their target genes through chromatin interactions.

We also found that in six of the 11 tissues, tissue-specific eQTLs are significantly enriched in tissue-specific FIREs. This result suggests that tissue-specific eQTLs may function through chromatin interactions that are also tissue specific, at least in those six tissues. However, lung showed a significant negative association. This might be due to more complicated mechanisms in lung, or heterogeneity in sample sources, location, or tissue cell types because we matched the two sources of data simply by tissue name. The potential heterogeneity might have introduced noise in our analyses, and some of our results might have been stronger if the data had been more homogeneous.

The relationship between tissue-specific eQTLs and tissue-specific chromatin interactions is helpful for identifying genes regulated by eQTLs through chromatin interactions in the corresponding tissue. For example, the brain cortex tissue DLPFC has 2954 tissue-specific eQTLs and 323 tissue-specific FIREs. When both factors were considered, we identified 32 DLPFC-specific eQTLs located in those DLPFC-specific FIREs. These eQTLs are significantly associated with 4 genes, including *ADGRB2* (adhesion G protein-coupled receptor B2), *WASF3* (WAS protein family member 3), *SPEF2* (sperm flagellar 2), and *XPA* (xeroderma pigmentosum complementation group A). Among these genes, *ADGRB2*, which encodes a transmembrane signaling receptor [25], has a brain-specific developmental expression pattern and its expression level is increased as the development of the brain progresses [26]. The TSS of this gene (chr1:32,192,718) is ~47Kb from a DLPFC-specific FIRE (chr1:32,240,000-32,320,000).

Due to the relatively low depth in the Hi-C data currently available for multiple tissues, we performed our analyses at the 40Kb resolution to avoid data sparsity. It would be ideal to perform the analyses at a higher resolution. However the availability of high-resolution Hi-C data for tissues is currently very limited.

The eQTL results used in our study were available only for SNP-gene pairs that are within 1 Mb distance. The power of the eQTL analysis is largely determined by sample size. Because of these issues, we might have missed some SNP-gene associations in our analyses. In addition, our TAD enrichment analysis did not account for linkage disequilibrium (LD) between eQTLs. The effects of LD, if any, are probably canceled out between the real and simulated datasets.

## Conclusions

In summary, we have demonstrated the close spatial proximity between eQTLs and their target genes across multiple human primary tissues. These results help us further understand the complementary effects of chromatin interactions and eQTLs in gene regulations.

## Methods

### Data description

We used Hi-C and eQTL data of 11 primary human tissues and 2 cell lines from Schmitt et al. [23] and the GTEx project [3], including the lymphoblastoid cell line GM12878, the fetal lung fibroblast cell line IMR90, and adrenal, aorta, dorsolateral prefrontal cortex (DLPFC), hippocampus, left ventricle, liver, lung, ovary, pancreas, small bowel and spleen tissues (Additional file 1: Table S1). We focus on the autosomes in all our analyses. The reference genome is hg19.

The Hi-C data contained over 2.9 billion raw intra-chromosomal unique read pairs on the 13 samples, out of which > 1 billion have distance >15Kb. We used 40Kb bin resolution for the Hi-C data. We also downloaded the information for TAD boundaries and FIREs from Schmitt et al. [23]. On average, there are 2068 TADs and 3681 FIREs per sample. Schmitt et al. [23] study also published RNA-seq data for each tissue sample, measured by FPKM values. In the GTEx study, all tested SNP-gene pairs were within 1 Mb distance [3]. Details of data preprocessing are in the Additional file 1: Supplementary Materials.

### Regression analysis of chromatin interaction frequency

We first evaluated the relationship between CIF and eQTL results using regression analysis. We mapped every SNP-gene pair tested in the GTEx study to a bin pair. SNP-gene pairs mapped to the same 40Kb bin were excluded from our analysis; that is, if a tested gene falls in one bin, it must have a corresponding SNP or eQTL in the other bin. We defined the following features for every bin pair (*i*, *j*), where *i* < *j* : 1) the CIF (*I*_*Hi* − *C*_), 2) the number of eGenes with TSS in bin *i* or bin *j* (*G*_*eGene*_), 3) the number of tested genes with TSS mapped to bin *i* (*G*_*i*_), 4) the number of tested genes with TSS mapped to bin *j* (*G*_*j*_), and 5) the genomic distance between bin *i* and bin *j* (*D*=| *i* − *j*| ). We focused on bin pairs that contain at least one tested gene (i. e., *G*_*i*_ + *G*_*j*_ > 0). Since in the GTEx study, all tested SNP − gene pairs were within 1Mb distance, the bin pairs in our analysis also had distance ≤ 1Mb. In addition, because the samples in the two original studies came from different tissue sources, we focused on genes that expressed in both sources, specifically, genes that were tested in the GTEx study [3] and had FPKM> 1 in the corresponding tissue or cell line in Schmitt et al. [23].

We performed negative binomial regression of CIF on the number of eGenes in each tissue or cell line. Since the CIF between two loci is known to be affected by their genomic distance [16], we included distance as a covariate in our model:

1 $$ \ln \left({I}_{Hi-C}\right)\sim {G}_{eGene}+D $$

In addition, it is known that chromatin interactions are less frequent between a gene-dense compartment and a gene-poor compartment than those within the same compartment [16]. While the compartments are defined on multi-Mb scale, this result indicates that gene density may play a role in Hi-C interaction. We thus further adjusted for the unevenness in the distributions of genes between two bins. Specifically, we added *G*_*Diff*_ =  ∣ *G*_*i*_ − *G*_*j*_∣ to our model as another covariate:

2 $$ \ln \left({I}_{Hi-C}\right)\sim {G}_{eGene}+{G}_{Diff}+D $$

We also conducted stratified analyses over subsets stratified by *G*_*Diff*_.

In Results we showed that there were significant positive associations between CIF and the number of eGenes. To further evaluate whether these associations are truly due to eGenes, we repeated the regression analysis for the number of non-eGenes (defined as tested genes without any eQTLs), and compared its effect with the effect of eGenes. We also performed a regression analysis for the number of not expressed genes (defined as genes that had FPKM ≤ 1 in Schmitt et al. [23] and not tested in GTEx in the corresponding tissue or cell line). Specifically, we performed the following regression analyses:

3 $$ \ln \left({I}_{Hi-C}\right)\sim {G}_{non- eGene}+{G}_{Diff}+D $$

4 $$ \ln \left({I}_{Hi-C}\right)\sim {G}_{not\ expressed\ gene}+{G}_{Diff}+D $$

where *G*_*non* − *eGene*_ is the number of non-eGenes and *G*_*not expressed gene*_ is the number of not expressed genes.

We also performed sensitivity analysis by fitting alternative regression models where the input variables enter the models on the log scale or as categorical variables (details in Additional file 1: Supplementary Materials, and in Additional file 2: Additional Results).

### Enrichment analysis of eQTL-gene associations in TADs

We next evaluated if eQTL-gene associations are enriched in TADs for all the tissues and cell lines we considered. For each tested SNP-gene pair, we created a matched pseudo pair as a control: we kept the gene’s TSS position but flipped the position of SNP to be on the opposite side of the TSS but with the same distance from the TSS. For example, if the SNP is 93Kb downstream of the TSS, the flipped position will be 93Kb upstream of the TSS. The real SNP-gene pairs and the pseudo SNP-gene pairs have the same overall distribution of gene locations and same overall distribution of SNP-TSS distance. If the flipped position fell outside of the chromosome, both the real and the matched pseudo pair were removed from analysis.

We categorized SNP-gene pairs by two features: whether the pair is a real pair and whether the SNP and the gene’s TSS are in the same TAD. We then performed McNemar’s test on the resulting 2 × 2 table to detect whether there was a significantly higher probability for SNP-gene pairs to change from being inside the same TAD to falling in different TADs after flipping the position of SNP than the opposite change. We also performed Fisher’s exact test to evaluate the association between these two features.

In addition, we took distance into account by performing logistic regression *Y*~*X* + *D*, where *Y* indicates whether a SNP-gene pair is in the same TAD, *X* indicates whether the pair is real, and *D* is the distance between the SNP and the TSS of the gene. We also performed stratified analyses by stratifying the data by genomic distance ranging from 40Kb to 1 Mb.

### Analysis of tissue-specific FIREs and tissue-specific eQTLs

Finally, we investigated the tissue specificity of Hi-C data. We defined tissue-specific FIREs and studied their enrichment surrounding genes and their association with tissue-specific eQTLs. For each of the 11 tissues, we defined tissue-specific FIREs as those FIREs detected only in that tissue and not in any of the other 10 tissues. Tissue-specific eQTLs were similarly defined using the GTEx meta-analysis results (details in Additional file 1: Supplementary Materials). For GM12878 and IMR90, cell line-specific FIREs and eQTLs were similarly defined using all 13 samples we considered. For example, GM12878-specific FIREs are the FIREs detected only in GM12878 and not in any of the other 12 samples.

For each tissue, we examined whether tissue-specific eQTLs are enriched in tissue-specific FIREs. For each tissue and cell line, we counted the number of eQTLs according to whether the eQTL is tissue specific and whether it falls in a tissue-specific FIRE. We computed the odds ratio as the ratio of the fraction of tissue-specific eQTLs mapped to tissue-specific FIREs to the fraction of those mapped to the other FIREs of the tissue. We also computed Bonferroni corrected *p*-value and confidence interval at the 95% level after Bonferroni correction (i.e. 99.54% nominal level, where 0.9954 = 1–0.05/11).

## Additional files

Additional file 1: Supplementary description, tables, and figures. (DOCX 1137 kb)

Additional file 2: Additional results. (XLSX 18 kb)

## Abbreviations

*ADGRB2*: Adhesion G protein-coupled receptor B2
CIF: Chromatin interaction frequency
CRE: *Cis*-regulatory element
DLPFC: Dorsolateral prefrontal cortex
eQTLs: Expression quantitative trait loci
FIRE: Frequently interacting region
FPKMs: Fragments per kilobase of exon per million reads
GTEx: Genotype-Tissue Expression study
Hi-C: High-throughput chromatin conformation capture
Kb: Kilobase
LD: Linkage disequilibrium
Mb: Megabase
SNP: Single nucleotide polymorphism
*SPEF2*: Sperm flagellar 2
TAD: Topologically associating domain
*WASF3*: WAS protein family member 3
*XPA*: Xeroderma pigmentosum complementation group A
