# Supplementary material for: Joint analyses of multi-tissue Hi-C and eQTL data demonstrate close spatial proximity between eQTLs and their target genes
Source: BMC Genet. 2019 Apr 30;20:43. doi: 10.1186/s12863-019-0744-x (PMC6492392; doi:10.1186/s12863-019-0744-x)
Supplement: Supplementary file 1 — Supplementary description, tables, and figures. (DOCX 1137 kb) [file 12863_2019_744_MOESM1_ESM.docx]

**Supplementary Information**

**Joint analyses of multi-tissue Hi-C and eQTL data demonstrate close spatial proximity between eQTLs and their target genes**

Jingting Yu^1^, Ming Hu^2,^ * and Chun Li^1, 3,^ *

^1^ Department of Population and Quantitative Health Sciences, Case Western Reserve University, Cleveland, Ohio, United States of America

^2^ Department of Quantitative Health Sciences, Lerner Research Institute, Cleveland Clinic Foundation, Cleveland, Ohio, United States of America

^3^ Institute for Computational Biology, Case Western Reserve University, Cleveland, Ohio, United States of America

* Corresponding author

Email: [cxl791@case.edu](mailto:cxl791@case.edu) or [hum@ccf.org](mailto:hum@ccf.org).

**Part 1. Supplementary materials and methods**

**Data description**

We evaluated the relationship between chromatin interaction frequency and eQTL results using data collected from 11 human primary tissues and 2 cell lines. Specifically, Schmitt *et al*. [1] recently performed Hi-C experiments on 14 human primary tissues and 7 human cell lines. The GTEx consortium has generated eQTL data for 48 human primary tissues [2]. Between these two resources, 11 tissues and 2 cell lines overlap, including the lymphoblastoid cell line GM12878, the fetal lung fibroblast cell line IMR90, and adrenal, aorta, dorsolateral prefrontal cortex (DLPFC), hippocampus, left ventricle, liver, lung, ovary, pancreas, small bowel and spleen tissues (**Supplementary** **Table S1**).

From the Schmitt *et al.* study (GEO accession number: GSE87112), we downloaded chromatin interaction frequency, TAD boundaries and frequently interacting regions (FIREs), all at 40Kb resolution. We also obtained RNA-seq gene expression data for these samples (also from GSE87112). The gene expression levels were quantified as FPKMs based on the GENCODE (Version 19) annotation. For tissues with multiple samples or donors, the mean FPKM was calculated for each gene.

We also downloaded eQTL data from the GTEx Portal (Version 7): 1) statistically significant SNP-gene associations (file “GTEx_Analysis_v7_eQTL.tar.gz”); 2) all tested SNP-gene pairs (file “GTEx_Analysis_v7_eQTL_all_associations.tar.gz”); and 3) meta-analysis results (file “GTEx_Analysis_v7.metasoft.txt.gz”). According to the GTEx analysis procedures, all tested SNPs were located within 1Mb of the transcription start site (TSS) of the tested genes. A gene is called an eGene if it has at least one significantly associated SNP with <5% false discovery rate (FDR).

**Hi-C and eQTL data preprocessing**

For each tissue and cell line, we created the chromatin interaction matrices for long-range intra-chromosomal interactions for all autosomes. For each chromosome, we extracted intra-chromosomal read pairs and removed the pairs within 15Kb distance. The chromosome was divided into 40Kb bins. For each bin pair, we obtained chromatin interaction frequency by counting the number of read pairs mapped to the bins.

All SNP-gene pairs tested by GTEx [2] were mapped to 40Kb bin pairs so that the SNP is in one bin and the TSS of the tested gene is in the other bin. SNP-gene pairs that were mapped to the same 40Kb bin were excluded from our analyses.

**Identification of tissue-specific eQTLs**

Based on the GTEx meta-analysis results [2], we defined the tissue-specific eQTLs as those that were significant only in that tissue and not for any of the other 10 tissues. Specifically, the meta-analysis results contained the estimated probability (*m* value) that an eQTL has an effect on a gene in a tissue. Since a SNP could have effects on multiple genes in a tissue, we used the maximum *m* value for the SNP to represent its effect in that tissue. SNPs that were not tested in all 11 tissues were excluded. We then defined an eQTL to be specific to a tissue if its max(*m*) $>$0.9 for that tissue and its max(*m*) $\leq$0.5 for all of the other 10 tissues.

**Alternative negative binomial regression models**

In addition to the negative binomial models (1) and (2) described in the main manuscript, we also performed the following analyses:

$$\ln\left( I_{Hi-C} \right)\sim ln(G_{eGene}+0.01)+ln(D) \left( 5 \right).$$

Here the addition of a small constant, 0.01, is to ensure the log-transformation can be carried out. Similarly, we also fit another model:

$$\ln\left( I_{Hi-C} \right)\sim{ln(G}_{eGene}+0.01)+abs\left( \ln\left( G_{i} \right)-\ln\left( G_{j} \right) \right)+\ln\left( D \right) \left( 6 \right).$$

The results for models (5) and (6) are in Figure S3.

We also fitted model (2) by treating the number of eGenes as a categorical variable. Results are in Supplementary_AdditionalResults. The number of eGenes ranged from 0 to 8 for all the bin pairs we analyzed across all tissues and cell lines. The effects of the statistically significant categories are comparable with those from model (2).

**Distribution of distance for significant eQTL-gene pairs and Hi-C peaks**

We computed the distance between the eQTL and the TSS for all significant eQTL-gene pairs and between the bins of significant Hi-C peaks according to Fit-Hi-C analysis [3]. The distributions are very similar across tissues for each of the two data sources, and for every tissue, the distributions are quite comparable between the data sources (Figure S5).

**Part 2. Supplementary tables**

**Supplementary Table S1. Data summary for all tissues and cell lines we analyzed.**

|  |  | **Schmitt *et al.*** | | | | **GTEx project** | |
| --- | --- | --- | --- | --- | --- | --- | --- |
|  |  | **Number of read pairs (>15Kb)** | **Number of TADs** | **Number of FIREs** | **Number of genes with FPKM >1** | **Number of tested genes** | **Number of eGenes** |
| **Tissues** | Adrenal | 25,843,694 | 2,043 | 3,407 | 15,221 | 22,376 | 6,233 |
|  | Aorta | 106,069,045 | 1,939 | 3,359 | 14,349 | 22,366 | 8,918 |
|  | DLPFC | 30,731,911 | 2,075 | 3,444 | 15,257 | 23,244 | 4,903 |
|  | Hippocampus | 34,430,763 | 2,083 | 3,616 | 15,052 | 22,953 | 3,157 |
|  | Left ventricle | 145,013,101 | 1,975 | 2,892 | 14,075 | 20,155 | 7,177 |
|  | Liver | 134,867,731 | 2,162 | 3,296 | 13,733 | 21,053 | 3,886 |
|  | Lung | 25,773,838 | 2,136 | 3,961 | 16,371 | 24,470 | 11,005 |
|  | Ovary | 22,823,424 | 2,051 | 3,471 | 15,679 | 23,567 | 3,680 |
|  | Pancreas | 33,874,614 | 2,083 | 3,723 | 14,768 | 21,339 | 6,972 |
|  | Small Bowel | 23,384,857 | 2,081 | 3,505 | 14,900 | 24,492 | 4,602 |
|  | Spleen | 37,135,245 | 2,222 | 3,694 | 16,073 | 23,795 | 6,392 |
| **Cell lines** | GM12878 | 128,525,187 | 2,188 | 4,755 | 14,177 | 21,467 | 3,724 |
|  | IMR90 | 350,084,081 | 2,127 | 4,727 | 13,033 | 20,848 | 10,774 |

**Supplementary Table S2. Sample size for the stratification analysis by the difference in gene density.**

|  | **Difference in the number of tested genes between two bins** | | | | | | | | |
| --- | --- | --- | --- | --- | --- | --- | --- | --- | --- |
|  | 0 | 1 | 2 | 3 | 4 | 5 | 6 | 7 | 8 |
| **Adrenal** | 36652 | 308602 | 71250 | 14551 | 3977 | 1356 | 506 | 148 | 23 |
| **Aorta** | 33448 | 300673 | 68007 | 11860 | 3599 | 1059 | 224 | 51 |  |
| **DLPFC** | 36493 | 323891 | 69208 | 12922 | 3244 | 578 | 210 | 57 |  |
| **Hippocampus** | 35536 | 317555 | 68717 | 12976 | 3220 | 828 | 126 | 103 |  |
| **Left ventricle** | 32643 | 295399 | 65238 | 11256 | 3059 | 866 | 327 | 53 |  |
| **Liver** | 31971 | 293546 | 63667 | 10796 | 3113 | 972 | 205 | 24 |  |
| **Lung** | 40312 | 323878 | 75683 | 15547 | 4352 | 1286 | 315 | 86 | 34 |
| **Ovary** | 35873 | 309660 | 74355 | 15415 | 3790 | 1169 | 270 | 61 |  |
| **Pancreas** | 35315 | 303533 | 68884 | 13531 | 3938 | 1273 | 323 | 125 |  |
| **Small bowel** | 36237 | 307038 | 68595 | 13617 | 3498 | 1070 | 417 | 108 | 37 |
| **Spleen** | 37386 | 305019 | 76369 | 16598 | 4228 | 1458 | 523 | 99 | 27 |
| **GM12878** | 28676 | 271965 | 71859 | 13830 | 3414 | 1130 | 356 | 109 | 14 |
| **IMR90** | 28698 | 281463 | 59493 | 10660 | 2358 | 649 | 204 | 30 |  |

**Supplementary Table S3. Pearson correlation between the number of eGenes and the number of total tested genes.**

| Tissues | Correlation |
| --- | --- |
| Adrenal | 0.14 |
| Aorta | 0.17 |
| DLPFC | 0.10 |
| Hippocampus | 0.07 |
| Left ventricle | 0.17 |
| Liver | 0.10 |
| Lung | 0.20 |
| Ovary | 0.10 |
| Pancreas | 0.16 |
| Small bowel | 0.11 |
| Spleen | 0.13 |
| Cell lines |  |
| GM12878 | 0.10 |
| IMR90 | 0.18 |

**Part 3. Supplementary figures**


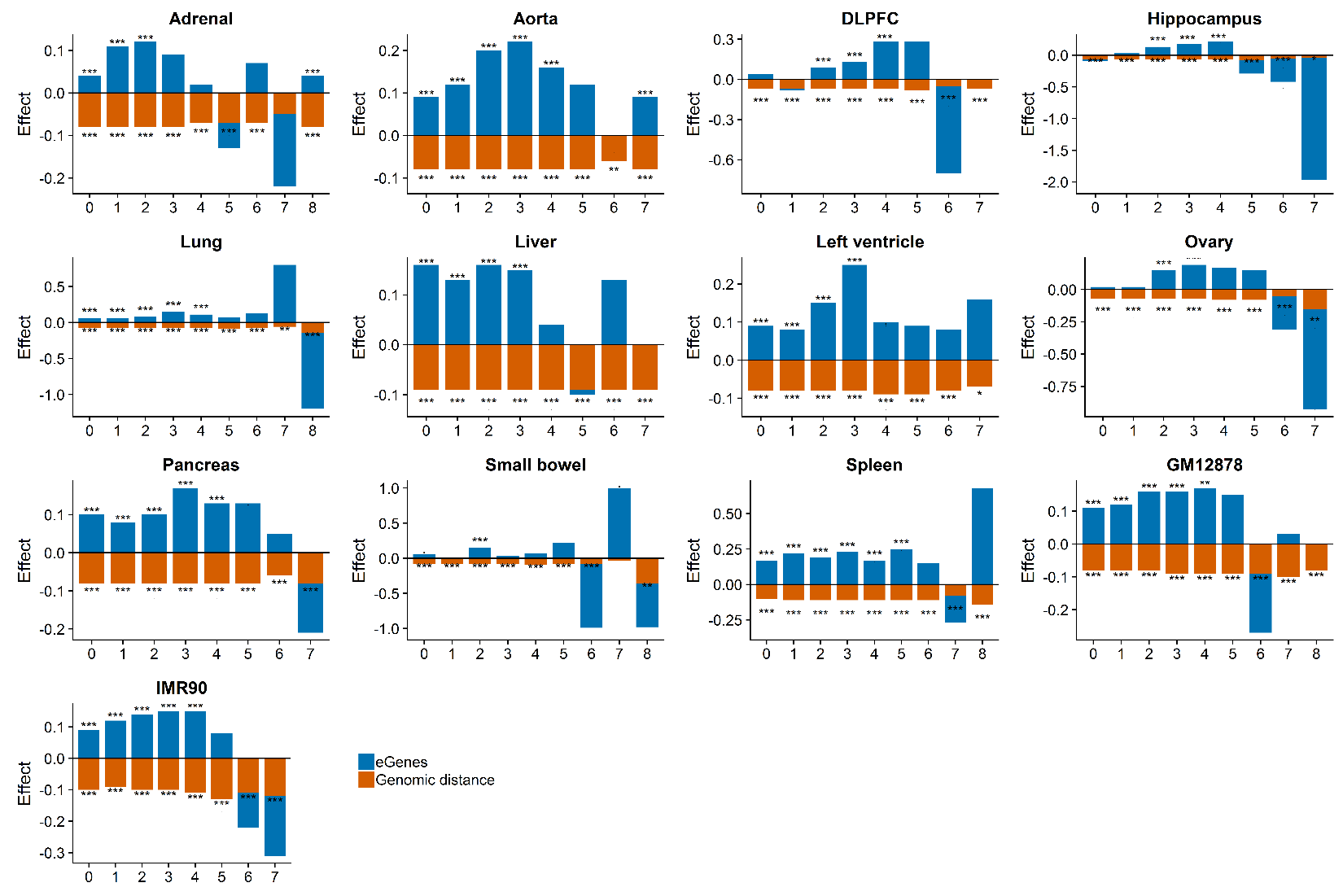


**Figure S1. Estimated effects of the number of eGenes and genomic distance on chromatin interaction frequency in stratified analyses.** For each tissue and cell lines, we stratified the bin pairs by the difference in gene density, and then fitted the negative binomial regression model (1) (see Methods) in each stratum. The estimated effect of the number of eGenes is in blue and that of genomic distance is in orange. (***)$P<0.001$; (**)$P<0.01$; (*)$P<0.05$; (.)$P<0.1$.


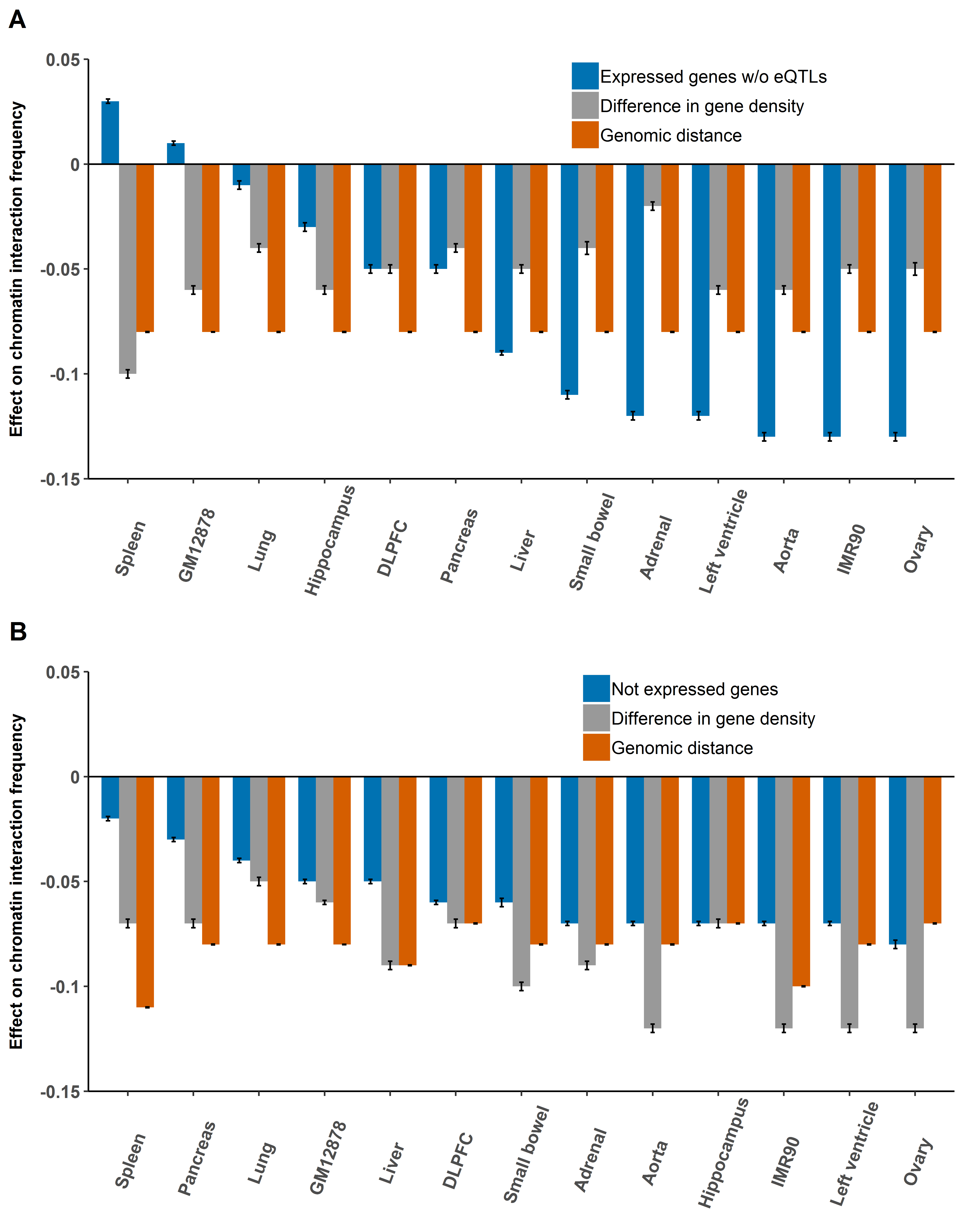


**Figure S2. Estimated coefficients from models (3) and (4).** The results for model (3) are in panel (A) and those for model (4) are in panel (B). The error bars are $\pm$ standard error.


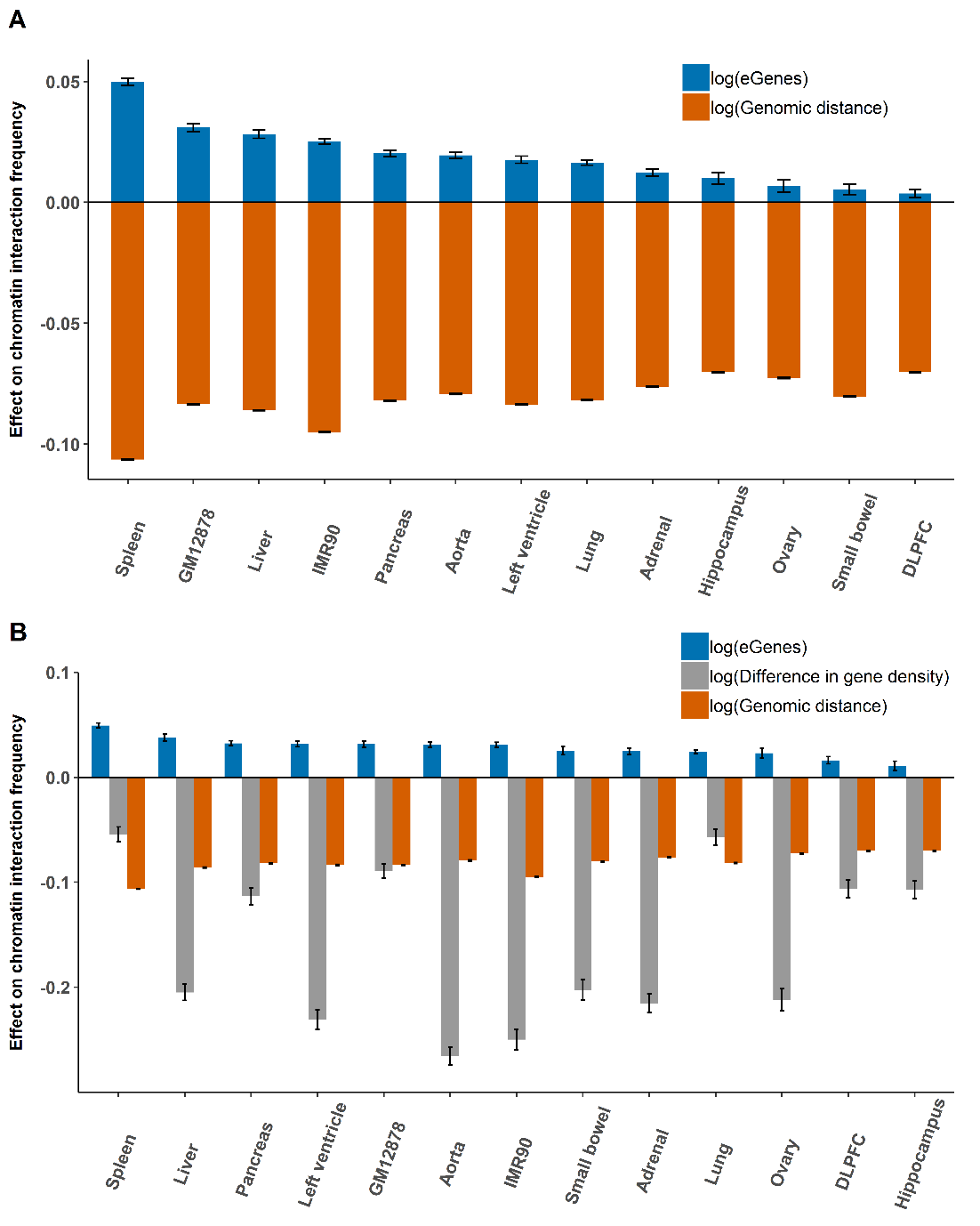


**Figure S3. Estimated coefficients from alternative models (5) and (6).** The results for model (3) are in panel (A) and those for model (4) are in panel (B). The error bars are $\pm$ standard error.


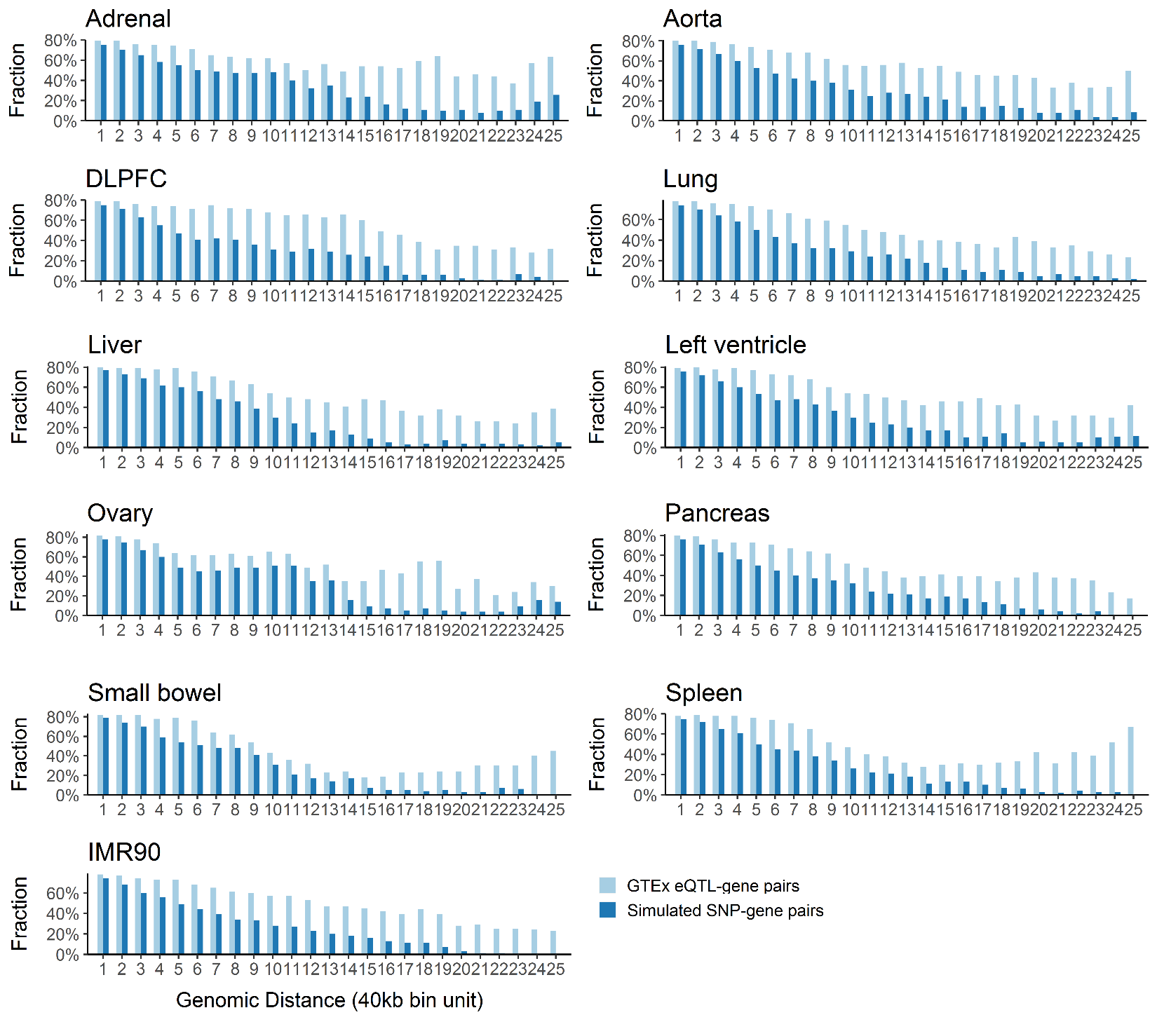


**Figure S4. Enrichment of eQTL-gene associations within TADs.** Similar to **Figure 3B**, for 10 other tissues (adrenal, aorta, DLPFC, lung, liver, left ventricle, ovary, pancreas, small bowel and spleen) and 1 cell line (IMR90). Detailed results after stratifying by the distance between SNP and TSS of target genes (x-axis, in 40Kb). All comparisons have p<0.001.


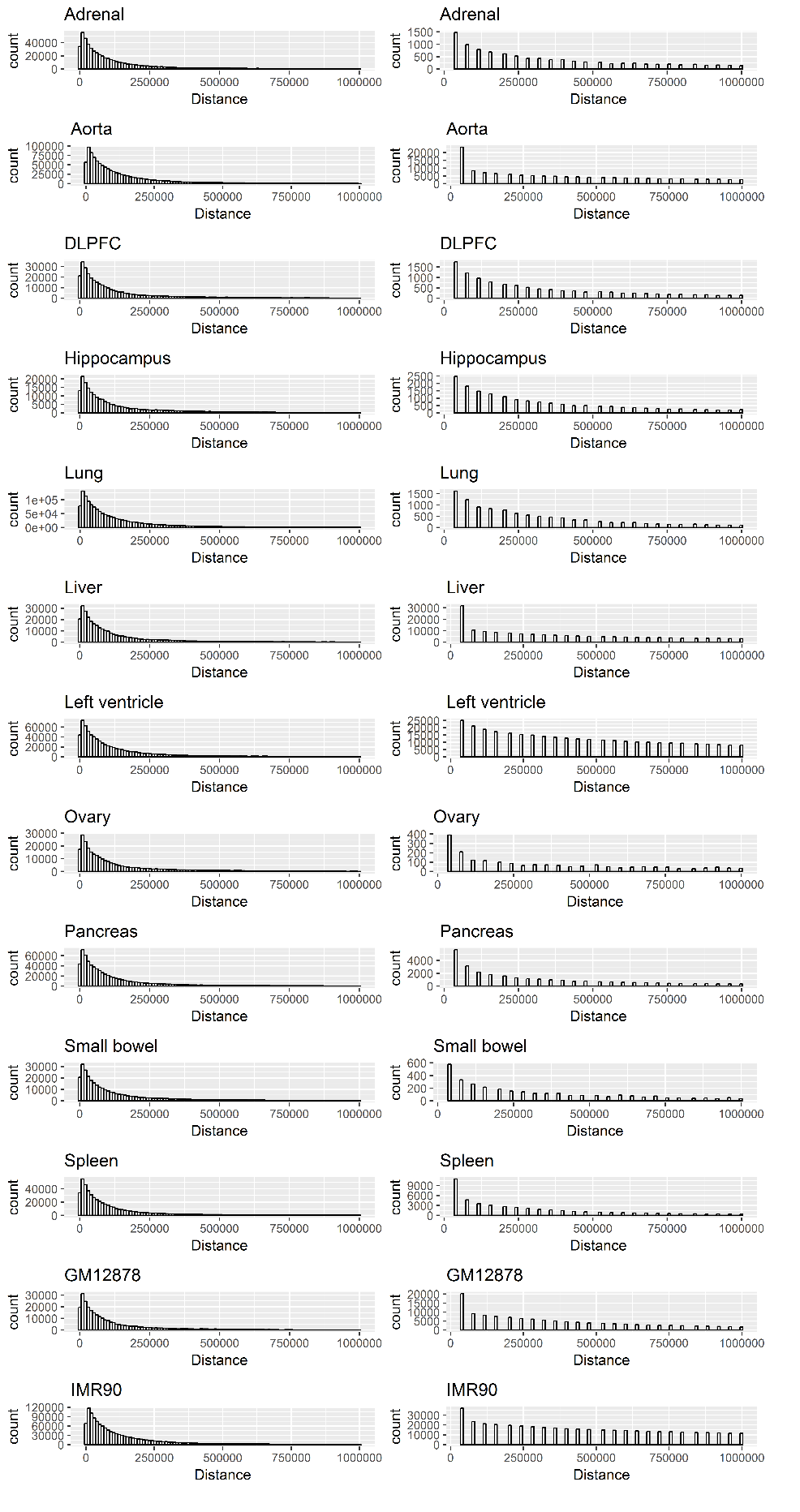


**Figure S5. Distributions of distance.** The left column is eQTL-gene pairs and the right column is Hi-C peaks.

**References**

1. Schmitt AD, Hu M, Jung I, Xu Z, Qiu Y, Tan CL, et al. A compendium of chromatin contact maps reveals spatially active regions in the human genome. Cell Rep. 2016;17(8):2042-59.
2. The Genotype-Tissue Expression (GTEx) pilot analysis: Multitissue gene regulation in humans. Science. 2015;348:648-60.
3. Ferhat Ay, Timothy L. Bailey, William S. Noble. 2014. "Statistical confidence estimation for Hi-C data reveals regulatory chromatin contacts." Genome Research. 2014;24(6):999-1011.
